# In cats undergoing midline ovariohysterectomy, is the use of local anaesthesia with bupivacaine associated with a reduction in postoperative pain score?

**DOI:** 10.18849/ve.v8i1.552

**Published:** 2023-02-22

**Authors:** Tara Freeman+, Amelia Wisbey+, Kate Burroughs+, Samantha Gentle+, Connie Ellis+, Sarah Batt-Williams+

**Keywords:** OVARIOHYSTERECTOMY, FELINE, BUPIVACAINE, LOCAL ANAESTHESIA, POSTOPERATIVE PAIN, PERIOPERATIVE ANALGESIA, NEUTERING, SPAY

## Abstract

**PICO question:**

In cats undergoing midline ovariohysterectomy, is the use of local anaesthesia with bupivacaine via intraperitoneal or subcutaneous injections, in comparison with the use of a control substance or other analgesic measure, associated with a reduction in postoperative pain score?

**Clinical bottom line:**

**Category of research:**

Treatment.

**Number and type of study designs reviewed:**

Three studies were critically appraised in this Knowledge Summary. They included two prospective, blinded, randomised, controlled clinical trials and one prospective randomised, blinded clinical trial.

**Strength of evidence:**

Weak.

**Outcomes reported:**

Bupivacaine does not eliminate postoperative pain in cats undergoing ovariohysterectomies; minimal evidence was found to suggest that it was better at reducing postoperative pain scores in comparison to other analgesics. However, bupivacaine may provide analgesic benefits to cats when administered via intraperitoneal or subcutaneous injections as local anaesthesia and in combination with other analgesic agents. The need for postoperative rescue analgesia was minimised when bupivacaine was administered prior to or during the ovariohysterectomy.

**Conclusion:**

When compared to a control, pain scores for the participating cats were lower after administration of bupivacaine, however, statistical significance was only reached in one of the studies. Additionally, other medications were found to lower the post operative pain score to a greater effect. However, bupivacaine administration is cheap and simple to perform, so it’s use as part of a multimodal analgesic protocol is supported. Confounding factors within the studies may have altered the perceived effectiveness of the analgesic properties of bupivacaine though, so further investigation involving larger cohorts with standardised controls would be prudent.

## How to apply this evidence in practice

The application of evidence into practice should take into account multiple factors, not limited to: individual clinical expertise, patient’s circumstances and owners’ values, country, location or clinic where you work, the individual case in front of you, the availability of therapies and resources.

Knowledge Summaries are a resource to help reinforce or inform decision making. They do not override the responsibility or judgement of the practitioner to do what is best for the animal in their care.

## Clinical scenario

A client is considering an ovariohysterectomy for their cat. They notice a fee for a bupivacaine local anaesthetic block and question the necessity of this. You review the evidence for the use of bupivacaine as part of a multi-modal anaesthetic protocol in cats undergoing elective ovariohysterectomy via ventral midline coeliotomy.

## The evidence

All three studies followed a reliable method, appropriate to the category of research.  Each of the three studies consistently found that bupivacaine provided some level of analgesia in comparison to the saline / placebo measures. However, they were inconclusive in verifying bupivacaine’s superiority over other analgesics.

Unvalidated pain scales were used in all three studies which may have resulted in inaccurate representation of participant pain. Although the study’s individual methods were appropriate, combined use of validated and unvalidated pain scales may have impacted results drawn from the studies. Whilst statistical significance was only reached in one of the three studies, trend to lower pain scores in cats receiving bupivacaine was noted in all three.

## Summary of the evidence

**Benito et al. (2016a):** 

Population:	Client-owned, healthy mixed-breed female cats undergoing ventral midline incision for ovariohysterectomy. Inclusion criteria: Client owned cats. Female. Healthy, based on medical history and complete physical examination and haematology. Exclusion criteria: Aggression. Cardiac arrhythmias. Pregnancy. Lactation. Obesity (body condition score of >7 of scale 1–9). Anaemia. Clinical signs of disease.
Sample size:	n = 45 cats.
Intervention details:	Intervention groups: The same volumes of bupivacaine or saline were administered via injection in each group: Saline 0.9% intraperitoneal, negative control group (NG) (n = 15). Saline 0.9% intraperitoneal and meloxicam subcutaneous, positive control group (PG) (n = 15). Bupivacaine hydrochloride (HCl) 0.25% intraperitoneal, bupivacaine group (BG) (n = 15). Group characteristics: PG: Mean body condition score 5 (range 4–5). Mean weight 3.1 (standard deviation [SD] 0.6). NG: Mean body condition score 5 (range 4–5). Mean weight 3.1 (SD 0.6). BG: Mean body condition score 5 (range 4–7). Mean weight 3.1 (SD 0.8). Premedication: Given intramuscularly Acepromazine (0.05 mg/kg). Buprenorphine (0.01 mg/kg). Administration of local anaesthetic / saline: Intraperitoneal injections. The solution was divided equally into three parts and administered into the right and left ovarian pedicles and the caudal uterus. Injections given immediately before the ovariohysterectomy. Pain scale used: Dynamic and interactive visual analog scale (DIVAS). UNESP-Botucatu multidimensional composite pain scale (MCPS). Statistical analysis: Demographic data for each group was analysed using one-way ANOVA or χ2 test. Temporal changes within a treatment group were analysed using the Cochran-Mantel-Haenszel test for ordinal scores followed by pairwise comparisons between groups.
Study design:	Prospective, blinded, randomised, controlled, clinical trial.
Outcome studied:	Requirement for rescue analgesia. Pain scores of the patients (before, at 30 minutes, 1 hour and 1.5 hours after surgery). Mean mechanical nociceptive thresholds (MNT) (before and at 2 hours after surgery.
Main Findings (relevant to PICO question)	Pain scoring: Postoperative pain scores increased compared to baseline (pre-operative) pain score in all treatment groups. DIVAS A statistically significant increase in postoperative pain score was found for all treatment groups when compared to baseline. PG at 0.5 hours (P = 0.002), 1 hour (P = 0.0008) and 2 hours (P = 0.0006). NG at 0.5 hours, 1 hour and 2 hours (P = <0.0001 at all intervals). BG at 1 hour and 2 hours (P = <0.0001 at all intervals). However, there was no statistically significant difference between DIVAS pain scores among treatment groups, suggesting that all treatments provided the same level of analgesia. MCPS A statistically significant increase in postoperative pain scores was found for all treatment groups when compared to baseline (pre-operative pain score). PG, NG and BG at 0.5 hours, 1 hour and 2 hours (P = <0.0001 for all intervals). However, there was no statistically significant difference between MCPS pain scores among treatment groups, again suggesting that all treatments provided the same level of analgesia. Lower DIVAS and MCPS scores were seen in the PG and BG groups compared to the NG group. No statistical significance was found though. Requirement of rescue analgesia: 18 cats required rescue analgesia (NG, n =12, 80%, PG, n =2, 13%, BG, n =4, 27%). There was a significant difference in need for rescue analgesia between NG and BG (P = 0.02). There was a significant difference in the need for rescue analgesia between NG and PG (P = 0.0004). There was no significance in need for rescue analgesia between the PG and BG (P = > 0.05).
Limitations:	Small sample size, possibly impacting generalisability of results and could lead to a type 2 error. The effects of sedation were not clear and may have affected the results. The DIVAS pain scale was not a validated pain scale for cats so may not be accurate in identifying levels of pain. The study was conducted in a veterinary teaching hospital but the level of experience of the veterinary surgeon was not stated. Inexperience could have led to sub-optimal technique and hindered the reliability of the results.

**Fudge et al. (2020):** 

Population:	Healthy female shelter cats undergoing ventral midline ovariohysterectomy. Inclusion criteria: Healthy. Female cats (from shelter). Weighed >0.9 kg. Age >2 months. Exclusion criteria: Pregnant. Incomplete data available.
Sample size:	n = 212 cats.
Intervention details:	Intervention groups: The bupivacaine or saline were injected into the suspensory ligaments and vessels, uterine body and incisional subcutaneous tissues for the treatment and placebo groups. Treatment (bupivacaine 0.5%, 2 mg/kg). Placebo control (0.9% saline). Sham control (observation only). Phase 1 36 cats (bupivacaine group n = 12, saline group n = 17, sham group n = 7). Phase 2 176 cats (bupivacaine group n = 59, saline group n = 41, sham group n = 76). Premedication: Buprenorphine (0.01 mg/kg) given intramuscularly. Induction: Intramuscular injection of ketamine and dexmedetomidine at the doses of; 15 mg ketamine + 0.0075 mg dexmedetomidine (cats weighing 0.9–1.8 kg), 20 mg ketamine + 0.01 mg dexmedetomidine (cats weighing > 1.8 kg). Administration of local anaesthetic: Bupivacaine (or saline) volume was equally divided into four. Injections into right (1) and left (2) suspensory ligament of ovary, mesovarium, and pedicles of ovarian vessels, uterine body (3), and incisional subcutaneous tissue (4). Administration occurred during ovariohysterectomy. Pain scale used: Modified UNESP-Botucatu multidimensional composite pain scale (MCPS) was used in the first phase. (Subscale 3 including arterial blood pressure and appetite monitoring was removed). 0–10 numerical rating scale (NRS) used in both phases. Modified Colorado State University Feline Acute Pain Scale (mCSU) was used in the second phase (removed palpation of the surgical site). Statistical analysis: One-way ANOVA was used to compare age, weight, and breed. One-way ANOVA was used to determine potential differences between pain score averages. A Holm-Bonferroni post-hoc analysis was carried out to compare groups where a difference was found via ANOVA. A correlation analysis was used to measure the strength of the relationship between evaluators scores.
Study design:	Prospective, randomised, double-blinded, placebo-controlled clinical trial. The study consisted of two phases.
Outcome studied:	Pain score of the patients, 1 hour post anaesthesia recovery and immediately before the same-day discharge (<7 hours post anaesthesia). Requirement for rescue analgesia.
Main Findings (relevant to PICO question)	After standardisation and comparison, bias in the agreement between the evaluators’ pain scores was found in the first phase (-0.15), with NRS scores higher than MCPS. Data gathered from phase one was disregarded, therefore, the below results are from phase two only (176 cats). Bias was smaller in the second phase (0.02) and was deemed acceptable. Pain scores: As participants were randomly allocated, there was natural variation leading to, significant differences in age and weight between the groups, so results were separated into different categories for each treatment group during data analysis, this was to help with interpretation. Weight group 0.9–1.5 kg and >1.5–2.7 kg: no significant difference in the pain scores among the three groups in this weight category. Weight group >2.7 kg: pain scores for the bupivacaine group were significantly lower than in the control groups at 1 hour post-recovery (P = 0.008) and at discharge (P = 0.004). For all weight groups and drug groups, pain scores were significantly higher 1 hour post-recovery than at discharge. Requirement for rescue analgesia: None of the cats required rescue analgesia.
Limitations:	Pain was quantified via a numerical rating scale (NRS); a unidimensional scale which does not consider the complexity of the pain experience. However, these scores were compared to a multidimensional scale mCSU which would have minimised interpretation issues. The pain scales used were not validated for use in cats and were modified by the researchers to suit the data they could collect. Participants in the saline group were significantly older, this may have impacted their metabolism of the treatments, possibly altering postoperative pain scores. Induction of anaesthesia with known analgesic drugs (ketamine and dexmedetomidine) did not account for the current weight of the animal (only dependent on more or less than 1.8 kg). So that heavier patients received quantitatively less systemic analgesics when reported to their body weight (or surface area) than lighter patients. This may have blunted pain scores and decreased differences between groups in the first two weight strata.

**Tobias et al. (2006):** 

Population:	Client-owned, healthy female cats undergoing midline ovariohysterectomy. Inclusion criteria: Healthy (ASA1). Intact females. Weighing over 2.2 kg. Unknown ages but estimated at least 6 months old. Exclusion criteria: Pregnant.
Sample size:	n = 52 cats.
Intervention details:	Intervention groups: Two cats were removed from the study after surgery because they had anemia due to accidental overdose of intravenous fluids, leaving the sample size at 50 cats, organised into the following groups. Carprofen (2.2 mg/kg) PO before sedation (n = 12). Ketoprofen (2.2 mg/kg) SC after sedation (n = 14). Butorphanol tartrate (0.44 mg/kg) IM after sedation (n = 12). Bupivacaine (1.1 mg/kg) SC before first surgical incision (n = 12). Premedication: Acepromazine (0.022 mg/kg) intramuscularly (IM). Ketamine (4.4 mg/kg) IM. Administration of local anaesthetic: Injected subcutaneously over a 2.5 cm distance along the midline, midway between the umbilicus and pubis. Pain scales used: Visual analog scale (VAS). Interactive visual analog scale (IVAS). Statistical analysis: Mixed model analysis of variance procedure to evaluate pain scores where cat was included in the model as a random factor.
Study design:	Prospective, randomised, blinded clinical trial.
Outcome studied:	Patient pain scores. Taken with VAS and IVAS preoperatively and postoperatively (extubation and 1, 2, 4, 8, 12 and 24 hours after surgery). Requirement for rescue analgesia. Mean cortisol concentrations in blood plasma preoperatively and 1, 2, 4, 8, 12 and 24 hours postoperatively. Mean plasma concentrations of the drug in the blood plasma preoperatively and 1, 2, 4, and 8 hours postoperatively. The results mentioned in points 3 and 4 are irrelevant to the PICO question so there will be no further mention of them in this Knowledge Summary.
Main Findings (relevant to PICO question)	Pain scoring: All cats had VAS and IVAS scores of 0 before surgery (baseline). Cats receiving bupivacaine had significantly higher VAS and IVAS pain scores at 1 and 2 hours post-surgery compared to baseline, as did those receiving carprofen and ketoprofen (P ≤ 0.0122). In all groups, cats’ VAS and IVAS scores were not significantly different to baseline at 4–24 hours after surgery. Pain scores for cats receiving bupivacaine were significantly higher 1 hour post-surgery (P = 0.002) compared to those receiving butorphanol (P = 0.029). There was no significant difference in pain scores at any time between cats receiving carprofen, ketoprofen, or butorphanol. Rescue analgesia: Rescue analgesia was administered 2 hours after surgery for one patient in the bupivacaine infusion block group and 1 hour after surgery in one patient in the ketoprofen group.
Limitations:	One cat was given the wrong analgesic, so the groups were not evenly distributed, hindering comparison between groups. The varying sedative effects of the medications used was not accounted for in addition to other affects such as delayed onset of medications, this may have impacted the accuracy of pain scoring. It is unknown if the intravenous fluid therapy provided during surgery affected plasma concentrations of the medications, possibly impacting their effect on nociception. There was no mention of a sample size calculation and the sample size was small, hindering the generalisability of the study.

## Appraisal, application and reflection

Three studies were found to be appropriate for this Knowledge Summary. They were all published in peer-reviewed journals and used prospective, controlled clinical trials. Benito et al*.* (2016a) and Tobias et al*. *(2006) implemented a blinded randomised method and Fudge et al. (2020) used a randomised double blinded placebo-controlled method. All three studies had approval from relevant bodies and the researchers ensured that all participants were provided additional rescue analgesia postoperatively, if required.

The first study analysed was Benito et al*.* (2016a), which included 45 client-owned cats of varying breeds. The use of client-owned participants increased the applicability of findings to general practice.

The requirement for rescue analgesia was significantly higher in the group receiving saline only compared to the groups receiving saline and meloxicam (P = 0.0004) or bupivacaine (P = 0.02). Of the saline group, 12/15 (80%) required further analgesia, compared to only 2/15 (13%) of the saline and meloxicam group, and 4/15 (27%) of the bupivacaine group. Whilst this shows that bupivacaine reduced patients’ pain scores and the need for further postoperative analgesia compared to the control, there was no statistically significant difference between the effectiveness of saline and meloxicam and the bupivacaine groups, suggesting that either protocol is appropriate.

Fudge et al*.* (2020) studied 212 cats from a shelter. Although a randomised study, the results were separated into different weight categories for each treatment group during data analysis to help with interpretation. All patients had an elevated pain score 1 hour postoperatively compared to discharge pain scores. Cats which received bupivacaine showed reduced pain scores compared to the two control groups (saline and sham controls) in all the weight brackets, however, the only statistical significance was found in the group weighing over 2.7 kg. It was found that cats both in the higher weight category and the bupivacaine group had a significantly lower pain score compared to the control groups 1 hour postoperatively (P = 0.008) and at discharge (P = 0.004). This implies that the effectiveness of bupivacaine may not be generalisable to all individuals due to weight related drug variations. However, the drug dosages were not specific to the patients’ exact weight, only to their weight category. This may have altered the significance of any difference in pain scores between the lower weight categories, and therefore should be considered when interpreting the significant data.

The third study, conducted by Tobias et al. (2006), assessed 52 client-owned cats. No sample size calculation was undertaken, which should be noted when making generalised assumptions from this study.

Baseline pain scores were taken using both chosen pain-scoring methods, and then repeated at intervals postoperatively. Participants who were given carprofen, ketoprofen, or bupivacaine had a significant increase in their pain scores, compared to the baselines (P ≤ 0.0122). These were recorded by both scoring methods at 1 and 2 hours postoperatively. Comparatively, those participants who received butorphanol had no significant change in their Visual Analogue Scale (VAS) scores, only a significant difference in the Interactive Visual Analogue Scale (IVAS) pain score 2 hours postoperatively (P = 0.0231). Additionally, participants from the bupivacaine group had higher postoperative pain scores compared to the other groups. Whilst this was only high enough in one participant to require rescue analgesia, it does suggest that there might be more beneficial analgesics to consider.

In general, these three main studies concluded that bupivacaine may have a clinical effect as an analgesic drug to reduce postoperative pain scores, however, statistical significance was not found in every study. This suggests that there was a possibility of the correlation between a bupivacaine local anaesthetic block and a reduced pain score being due to chance and other medications may be equally or even more effective in controlling postoperative pain.

Despite the similar generalised conclusions in the three main studies, there were some discrepancies between their findings. This suggests that a definitive conclusion cannot be drawn from these studies alone, without further research. Inconsistencies between methods, discussed below, may have contributed towards these differences in results.

The location of the local anaesthetic block was noted as a key difference in methodology, varying between studies. Benito et al*. *(2016a) and Fudge et al*.* (2020) administered the local anaesthetic in specific intraperitoneal locations, whereas Tobias et al. (2006) administered bupivacaine subcutaneously. This will have impacted the effect of the drugs and resulted in different nerves being blocked, hindering evaluation of the analgesic properties of bupivacaine.

Another variation to the methodologies was the chosen dose and dilution of bupivacaine. These variations may have indirectly resulted in the effectiveness of dose and dilution being evaluated, rather than the analgesic properties of bupivacaine. Benito et al*.* (2016a) used the lowest dilution of bupivacaine out of the three studies analysed. This chosen dilution was supported by research conducted by Benito et al*.* (2016b). Whilst it was found to be safe, the difference in dilution may hinder accuracy of the comparison between the three studies. Benito et al. (2016a) suggests that there is no difference between the analgesic properties of bupivacaine and their control medications, however, their findings may be reflective of the dilution rather than the analgesic properties of bupivacaine. Therefore, this needs to be considered when evaluating the effect on resulting pain scores.

Additionally, the chosen premedications varied throughout the studies. The studies either used acepromazine or dexmedetomidine as their chosen sedative. The duration of action for these two drugs is up to 6 hours and 20–60 minutes respectively , according to Ramsey, 2017. However, the duration of action of dexmedetomidine varies between sources, Granholm et al. (2006) found that heart rate and respiratory rate were still affected by dexmedetomidine over 3 hours after administration, indicating prolonged effects. Both acepromazine and dexmedetomidine had a risk of interfering with pain scores due to extended analgesic and sedative effects, possibly hindering the ability to infer analgesic properties of bupivicaine.

The analgesic effects of buprenorphine and ketamine should also be considered. The duration of action of these two drugs is up to 6 hours (Ramsey, 2017) and 20–40 minutes with a 1–4 hour recovery (NOAH, 2021). The duration of action of buprenorphine means it would have been active during most of the pain scores, the cats may also still have been recovering from the effects of ketamine. Additionally, ketamine can cause abnormal behaviour during recovery (Ramsey, 2017), which could hinder the process of pain evaluation.

Whilst it is important to recognise and understand the impact of premedication drugs, each study followed a standardised method throughout their data collection, therefore, producing valid results. Furthermore, the use of an opioid combined with a sedative drug is a routinely used premedication protocol in general practice (Murrell & Ford-Fennah, 2020). The premedications used are standard, applicable to a real-life setting and thus appropriate as administered in these studies.

A fourth key difference affecting comparison between the methods of the studies were the pain scoring systems used (figure 1). Benito et al*. *(2016a) used the Dynamic and Interactive Visual Analogue Scale (DIVAS) and UNESP-Botucatu Multidimensional composite pain scale (MCPS. Fudge et al*. *(2020) started with the latter, however many of the participants were feral, reducing application to practice and the ability to fully complete the scale. They switched to the Modified Colorado State University Feline Acute Pain Scale (mCSU) and Numerical Rating Scale (NRS). This change in pain scoring system reduced the palpation of the surgical site, and therefore the potential for staff injury. Tobias et al*. *(2006) employed the Visual Analogue Scale (VAS) and Interactive Visual Analogue Scale (IVAS), allowing for numerical and observational assessment.

Despite repeated use within the veterinary industry (Bloor, 2017), most of the pain scales used are not validated for cats. This should be considered when evaluating the reliability of the results, as they may have led to an inaccurate representation of participant pain. Issues with the reliability of pain scales were present in Fudge et al (2020), where the first phase of the study was disregarded due to significant difference between NRS and MCPS pain scores. NRS scores were higher than MCPS scores. The differences in scores were deemed as bias and therefore produced unreliable data for the study. However, the second phase of Fudge et al (2020)’s study replaced the validated MCPS scale with the mCSU, an unvalidated scale for use in cats. Therefore, the results are based on two unvalidated pain scales, and the results from phase one suggest they may have produced inaccurately high pain scores. The supposed bias should have been noted to highlight the possible inaccuracies of an unvalidated pain scale. These inaccuracies may also be reflected in the results of Tobias et al (2006), as they also only used unvalidated pain scales during their study.

The blinded nature of the study designs is expected to reduce bias, allowing for honest observations, without any preconceptions or opinions swaying the results (Moustgaard et al., 2020). In addition, all assessors throughout the three studies were trained to conduct the pain scores, which may have improved interobserver agreement.

**Figure 1:** Table showing the pain scales used in the studies.

Abbreviation	Pain scale	Validated for feline patients	Method of assessment	Which studies used which scoring system?
(DIVAS)	Dynamic and interactive visual analogue scale	No	Visualising pain using 10cm line (each cm represents a score).	Benito et al. (2016a) & Fudge et al. (2020) started with MCPS but altered their method
(MCPS)	UNESP-Botucatu Multidimensional composite pain scale	Yes	Emotional and physical effects of pain. Multiple behavioural categories are scored, equaling an overall score.	
(mCSU)	Modified Colorado State University Feline Acute Pain Scale	No	Removed palpation of surgical site. Multidimensional scale which uses behavioural cues to assess pain.	Fudge et al*. *(2020)
(NRS)	Numerical Rating Scale	No	The observer assigns a score of pain from 0–10 (0 = no pain, 10 = high pain).	
(VAS)	Visual Analogue Scale	No.	Visualising pain using a 10 cm line (each cm represents a score). 0–10 scale (0 = no pain, 10 = high pain).	Tobias et al. (2006)
(IVAS)	Interactive Visual Analogue Scale	No	Visualising pain using a 10 cm line (each cm represents a score). 0–10 scale.	

The timing of pain scoring was the final methodology difference analysed. Bupivacaine has an initial onset time of 2 and 5 minutes, with full block normally occurring between 5–10 minutes (Grubb & Lobprise, 2020). All postoperative pain scores were taken after this onset time and within bupivacaine’s duration of action. However, within the study by Fudge et al. (2020), postoperative pain scores were taken 1 hour into recovery and at discharge. Discharge times ranged from 1.7–7 hours post anaesthesia; therefore, some scores would have been taken close to the end of bupivacaine's duration of action, possibly reducing analgesic effects. This could have influenced the ability of pain scores to accurately assess the effect of bupivacaine at this timepoint.

Since evaluation of these three studies, Fudge et al. (2021) has published further research, comparing the use of bupivacaine with other targeted intraoperative injections (bupivacaine-lidocaine-epinephrine, dexamethasone, meloxicam). They conducted a prospective, randomised, double-blinded clinical trial with 151 cats, all undergoing midline ovariohysterectomies. The research followed similar guidelines to Fudge et al. (2020) but aimed to see if other drugs administered as targeted injections provided more effective analgesia than bupivacaine, like Tobias et al. (2006). Using the 0–10 Numerical Rating Scale (NRS), they found no statistical significance in postoperative pain scores between any of the groups 1 hour post-anaesthesia. Whilst meloxicam showed lower post-operative pain scores at 3 hours post-anaesthesia compared to all groups, it only gained statistically significant lower scores than the bupivacaine-lidocaine-epinephrine group (*P =* 0.018). Fudge et al. (2021) concluded that all of the tested drugs performed similarly as part of multimodal analgesia for feline ovariohysterectomies, except for meloxicam which may lower pain scores more than the bupivacaine-lidocaine-epinephrine block.

Similarly, Benito et al. (2019) conducted a study following Benito et al. (2016a), aiming to determine if administering bupivacaine with dexmedetomidine would provide superior analgesia in comparison to bupivacaine alone, when given to cats via splash block during ovariohysterectomy. This time Benito et al. (2019) used the UNESP-Botucatu composite pain scale to evaluate postoperative pain. They found that median pain scores in cats receiving just bupivacaine were significantly higher than those receiving bupivacaine and dexmedetomidine (P = 0.023) at 12 hours post-surgery, suggesting that administering bupivacaine along with another analgesic may reduce postoperative pain scores. Prior to this study, Benito et al. (2018) investigated the efficacy and pharmacokinetics of bupivacaine given in combination with epinephrine or dexmedetomidine to cats undergoing ovariohysterectomies. Results found that both drug combinations provide similar analgesic effects.  Although this study does not fit the PICO question for this Knowledge Summary due to the absence of testing bupivacaine alone, its conclusions support the use of bupivacaine in conjunction with another drug.

Furthermore, there may also be other alternative forms of bupivacaine which could be more favourable. Bupivicaine liposome injectable suspension is a longer lasting lipid based injectable that has a prolonged analgesic effect (Gordon-Evans et al., 2020). Although it is not directly comparable to bupivacaine hydrochloride and is not yet available globally, it may be another option to explore in the future.

Generalised findings conclude that a bupivacaine local anaesthetic block may influence post-operative pain in cats undergoing ovariohysterectomies, reducing the need for rescue analgesia. The technique of administering bupivacaine requires minimal skill to perform and is cost effective (Fudge et al., 2020).  However, further research is required to assess a range of other medication combinations, to ensure that the method is not only used because it is cheap and easy, but also effective and warranted. Research suggests that use of bupivacaine in conjunction with other analgesics may be preferable, but this should be explored further. Use of validated pain scales such as the Glasgow composite measure pain scale (WSAVA, 2015) and the incorporation of an objective assessment method such as the mechanical nociceptive threshold probe used by Benito et al. (2016a) could improve future research, although may be challenging with more aggressive patients. Conducting prospective randomised double blinded placebo-controlled clinical trials on different analgesics and locations could fill an evidence gap currently present.

## Methodology

**Search Strategy:** 

Databases searched and dates covered:	CAB Abstracts (2006–2022) PubMed (2006–2022) Science Direct (2006–2022)
Search strategy:	CAB Abstracts: (cat OR cats OR feline OR felines OR queen OR queens) (spay OR spey OR spaying OR speying OR ovariohysterectomy OR neutering OR neuter) (block OR blocking OR anaesthesia OR anaesthetic OR anesthesia OR anesthetic) (bupivacaine or bupivicaine) Post-operative OR postoperative OR pain scoring OR pain score Or Pain scale 1 AND 2 AND 3 AND 4 AND 5 PubMed: (feline OR cat OR cats OR queen OR queens OR felines) AND (spay OR spey OR spaying OR neutering OR neuter OR ovariohysterectomy) AND (local blocks OR local blocking OR local anaesthesia OR local anaesthetic OR local anesthesia OR local anesthetic) AND (bupivacaine OR bupivicaine) AND (post-operative pain score OR pain score OR pain scale) Science Direct: (feline) AND (spay OR ovariohysterectomy) AND (local anaesthesia) AND (bupivacaine) AND (post-operative pain score)
Dates searches performed:	09 Sep 2022

**Exclusion / Inclusion Criteria:** 

Exclusion:	Studies including species other than feline. Studies evaluating the effect of bupivacaine and the percentage of volatile agent used. Studies using ovariectomy or flank spay. Studies using combination blocks. Studies using a combination of local analgesic drugs. Studies published before 2006.
Inclusion:	Studies involving feline midline ovariohysterectomy. Studies bupivacaine administration and control measures (placebo or other analgesia). Peer-reviewed studies.

**Search Outcome:** 

Database	Number of results	Excluded – Canine / dog	Excluded – Ovariectomy	Excluded – Before 2006	Excluded – Irrelevant to PICO	Total relevant papers
CAB Abstracts	13	0	0	0	10	3
PubMed	7	0	0	0	4	3
Web of Science	53	47	1	1	3	1
Total relevant papers when duplicates removed						3

### Author contributions

**Tara Freeman:** Conceptualisation, Investigation, Resources, Writing – Original draft, Writing – Review & Editing (lead). **Amelia Wisby:** Conceptualisation, Investigation, Resources, Writing – Original draft. **Kate Burroughs:** Conceptualisation, Investigation, Resources, Writing – Original draft. **Samantha Gentle:** Conceptualisation, Investigation, Resources, Writing – Original draft. **Connie Ellis:** Conceptualisation, Investigation, Resources, Writing – Original draft. **Sarah Batt-Williams:** Supervision.

### ORCID

Tara Freeman: https://orcid.org/0000-0002-5588-9623

Kate Burroughs: https://orcid.org/0000-0003-4062-2739

Connie Ellis: https://orcid.org/0000-0002-3305-6866

Sarah Batt-Williams: https://orcid.org/0000-0001-8713-6944

### Conflict of Interest

The authors declare no conflicts of interest.
